# Whole Genome Sequencing Reveals the Effects of Recent Artificial Selection on Litter Size of Bamei Mutton Sheep

**DOI:** 10.3390/ani11010157

**Published:** 2021-01-12

**Authors:** Yaxin Yao, Zhangyuan Pan, Ran Di, Qiuyue Liu, Wenping Hu, Xiaofei Guo, Xiaoyun He, Shangquan Gan, Xiangyu Wang, Mingxing Chu

**Affiliations:** 1 Key Laboratory of Animal Genetics, Breeding and Reproduction of Ministry of Agriculture and Rural Affairs, Institute of Animal Science, Chinese Academy of Agricultural Sciences, Beijing 100193, China; yaoyaxin0602@126.com (Y.Y.); pzq170450077@163.com (Z.P.); dirangirl@163.com (R.D.); qiuyue1983921@163.com (Q.L.); pinkyhoho@163.com (W.H.); guoxfnongda@163.com (X.G.); hedayun@sina.cn (X.H.); 2 Tianjin Institute of Animal Sciences, Tianjin 300381, China; 3 State Key Laboratory of Sheep Genetic Improvement and Healthy Production, Xinjiang Academy of Agricultural and Reclamation Sciences, Shihezi 832000, China; shangquangan@163.com

**Keywords:** Bamei mutton sheep, whole-genome sequencing, litter size, selection signal analysis, breeding

## Abstract

**Simple Summary:**

Bamei mutton sheep is a Chinese domestic sheep breed developed by crossing German Mutton Merino sheep and indigenous Mongolian sheep for meat production. There is large variation in the reproductive abilities of Bamei mutton sheep. After recent artificial selection, the average lambing rate of the Bamei mutton nucleus group was over 150%. We used the FST (Fixation Index) and XP-EHH (The Cross-Population Extended Haplotype Homozygosity) statistical approach to detect the selective sweeps between high- and low-fecundity Bamei mutton sheep groups. *JUN* (*JUN* proto-oncogene, AP-1 transcription factor subunit), *ITPR3* (inositol 1,4,5-trisphosphate receptor type 3, *PLCB2* (phospholipase C beta 2), *HERC5* (HECT and RLD domain containing E3 ubiquitin protein ligase 5), and *KDM4B* (lysine demethylase 4B) were detected that are potential responsible for litter size. These observations provide a new opportunity to research the genetic variation influencing fecundity traits within a population evolving under artificial selection.

**Abstract:**

Bamei mutton sheep is a Chinese domestic sheep breed developed by crossing German Mutton Merino sheep and indigenous Mongolian sheep for meat production. Here, we focused on detecting candidate genes associated with the increasing of the litter size in this breeds under recent artificial selection to improve the efficiency of mutton production. We selected five high- and five low-fecundity Bamei mutton sheep for whole-genome resequencing to identify candidate genes for sheep prolificacy. We used the FST and XP-EHH statistical approach to detect the selective sweeps between these two groups. Combining the two selective sweep methods, the reproduction-related genes *JUN*, *ITPR3*, *PLCB2*, *HERC5*, and *KDM4B* were detected. *JUN*, *ITPR3*, and *PLCB2* play vital roles in GnRH (gonadotropin-releasing hormone), oxytocin, and estrogen signaling pathway. Moreover, *KDM4B*, which had the highest FST value, exhibits demethylase activity. It can affect reproduction by binding the promoters of estrogen-regulated genes, such as *FOXA1* (forkhead box A1) and *ESR1* (estrogen receptor 1). Notably, one nonsynonymous mutation (p.S936A) specific to the high-prolificacy group was identified at the TUDOR domain of KDM4B. These observations provide a new opportunity to research the genetic variation influencing fecundity traits within a population evolving under artificial selection. The identified genomic regions that are responsible for litter size can in turn be used for further selection.

## 1. Introduction

Sheep were the first grazing animals bred for their meat. Mutton still has major economic value for sheep production. The domestication of sheep was initiated approximately 9000 years ago in Southwest Asia, in present-day also in Iran and Turkey [1]. Sheep husbandry began 5000–5700 years ago in the Mongolian Plateau in China [2]. China has various sheep resources, including 42 indigenous sheep breeds [3]. These breeds are well adapted to the local plateau and desert environments [4], but the meat yield of these Chinese indigenous sheep breeds is so poor that it fails to meet the increasing consumer demand for mutton.

From the 1960s, in Bayannur of the Inner Mongolia Autonomous Region, German Mutton Merino sheep were imported and bred with indigenous Mongolian sheep to improve their meat yield. After 40 years of selection and improvement, in 2007, a novel breed, Bamei mutton sheep, was created and showed good genetic stability [5]. Under grazing conditions, Bamei mutton sheep is well adapted to dry and chilly winters in the Inner Mongolia Autonomous Region. Approximately 57,000 sheep of this type are raised mainly in this region. The lambs of Bamei mutton sheep grow faster than those of Small-Tail Han sheep. In terms of the live weight, they can reach about 53 kg at the age of 8 months and their average daily gain was found to be 199.54 g from 4 to 8 months of age under intensive feeding patterns [6]. Bamei mutton sheep are an important male breed in commercial hybrid sheep production in Bayannur [7]. The meat trait performance of this breed is excellent, but there is large variation in its reproductive abilities. The average lambing rate of the Bamei mutton nucleus group was over 150% [8]. The maintenance of high levels of fertility is vital for efficient sheep production [9]. Therefore, improving the level of fecundity of this new breed is a major focus of breeders.

Litter size has a major impact on desirable economic traits of sheep (e.g., meat, wool, and milk). Increasing litter size can improve the efficiency of sheep production [9]. The main factors affecting sheep fecundity include ovulation, uterine capacity, and placental efficiency. Litter size is a complex trait, but some major genes affecting prolificacy have been discovered in recent years. The *FecB* (bone morphogenetic protein 1B receptor, *BMPR1B*) gene was the first major gene found to affect prolificacy. Subsequently, many major genes, such as *FecX* (bone morphogenetic protein 15, *BMP15*), *FecG* (growth differentiation factor 9, *GDF9*), *FecL* (glycosylation enzyme beta-1,4-N-acetyl-galactosaminyltransferase 2, *B4GALNT2*), and *LEPR* (leptin receptor), were also reported [10,11].

As reported in *Science*, the first high-quality 2.61 Gb reference genomes of domestic sheep were sequenced and assembled in 2014. They can help in identifying genomic signatures of domestic traits in sheep [12]. By performing whole-genome resequencing on phenotypically divergent sheep populations, some selective sweeps were identified relating to important traits targeted by artificial selection during domestication, such as horn morphology, [1,13], coat color [14], tail morphology and fat deposition [3,15,16] and variation of thoracic vertebrae [17].

Regarding reproductive traits, using selection tests in pigs, previous studies demonstrated some strong selective signals belonging to the TGF-β signaling pathway [18,19]. In addition, in goats, the genes regulating seasonal reproduction and litter size have been specifically selected [20,21]. Moreover, some fecundity-related genes revealed a strong selection signature in sheep from Ethiopia and Europe [22,23].

Against this background, we performed whole-genome resequencing of 10 ewes selected to have extreme fecundity and sweeping analysis to identify the underlying variants and genes responsible for the litter size of Bamei mutton sheep under the influence of artificial selection.

## 2. Materials and Methods

### 2.1. Animals and DNA Preparation

In this study, we collected whole-blood samples from the jugular vein of 10 Bamei mutton sheep at the Xianghe Bamei mutton sheep-breeding park (Bayannur, China). These blood samples were placed in EDTA vacutainer tubes for storage. These ewes were about 3 years old and selected from among 500 sheep. Only ewes with litter size data showing that they had given birth more than three times were sampled. The selected ewes were grouped into two categories based on the phenotype of litter size (monotocous sheep giving birth to only one lamb in three consecutive parities and polytocous sheep giving birth to more than two lambs in two consecutive parities). Then, genomic DNA was extracted from 200 µL of sheep blood using a QIAamp DNA Blood Mini Kit (Qiagen, Hilden, Germany), in accordance with the manufacturer’s instructions. DNA quality and integrity were assessed by spectrophotometry (OD260/280) and 1.0% gel electrophoresis.

### 2.2. Genome Sequencing

High-quality DNA for Illumina sequencing library construction was randomly sheared into small pieces (300–400 bp). After end-repair, “A”-tailing and ligating to Illumina sequencing adapters, 400–500 bp ligated products were amplified by ligation-mediated PCR (LM-PCR). Then, 2 × 100 bp paired-end sequencing was carried out on an Illumina HiSeq 2500 sequencer and the original data were analyzed by Illumina HiSeq Control Software (Illumina, San Diego, CA, USA).

### 2.3. Read Processing and Variant Calling

NGS QC Toolkit v2.3.3 was used for quality control of the raw reads following three steps [24]. First, reads with >70% low-quality bases (score < 20) in the FASTQ files were filtered out. Second, reads containing N residues were filtered out. Third, low-quality ends (scores < 20) were trimmed. After this trimming, if the read length was <35, the read was removed. After this quality control, the reads of each sheep were mapped to the sheep genome assembly v3.1 (ftp://ftp.ncbi.nlm.nih.gov/genomes/all/GCA_000298735.1_Oar_v3.1/GCA_000298735.1_Oar_v3.1_genomic.fna.gz) using BWA v0.6.2 [25]. The .bam file was sorted by chromosome and duplicated reads were removed using SAMtools v0.1.19 [26].

Mapped reads of all samples were pooled for variant calling using SAMtools, with the parameters “mpileup–u–C50–DS–q20.” The .vcf file was generated by bcftools view with the parameter “-evcgN.” Then, vcfutils.pl with minimum depth “-d 20” and maximum depth “-D 300” was used to filter raw variants. Finally, the variants were annotated by ANNOVAR [27] version 2014-11-12, in accordance with Ensembl gene annotation (Oar_v3.1).

### 2.4. Population Genetics Analysis

The samples were separated into two groups (five monotocous individuals, five polytocous individuals). The SNP densities, minor allele frequencies, and Tajima’s D of each group were calculated by VCFtools v0.1.12b (https://vcftools.github.io/index.html) [28]. Only SNPs in autosomes were preserved for the phylogenetic analysis. A phylogenetic tree of all samples was generated using SNPhylo [29] version 20160204 (http://chibba.pgml.uga.edu/snphylo/), based on the maximum likelihood method. A total of 500,000 SNPs were randomly selected for calculating the linkage disequilibrium (LD) r^2^ using Haploview [30] with parameters set as follows: “--missingCutoff 0.2 --dprime --minMAF 0.1.” The SNP pairs were clustered based on the physical distances of these genes. The average LD (e.g., 0–1 kb) of each group was represented by the mean r^2^.

### 2.5. Selective Sweep Analysis

The pooled heterozygosity Hp was calculated over 10 kb windows using the formula:

$$Hp=2\times \frac{\sum n_{MAJ}\times \sum n_{MIN}}{{\left(\sum n_{MAJ}+\sum n_{MIN}\right)}^{2}}$$

where $\sum n_{MAJ}$ denotes the sum of major allele frequencies in a selected window and $\sum n_{MIN}$ denotes the sum of minor allele frequencies [31].

F_ST_ values were calculated between monotocous individuals and polytocous individuals for single SNPs using a method that adjusts for a small sample size [32]. We averaged F_ST_ values over 50 kb sliding windows along the genome with the Bio::PopGen::PopStats Package in BioPerl [33] and Z-transformed the resultant distribution. Putative selection targets were extracted from the extreme tail of the distribution by applying a Z(F_ST_) > 5 cut-off [34].

fastPHASE v1.4.0 was used to phase the genotypes of all samples with the parameters “-T10 –K8” [35]. Then, the phased data were used to calculate the cross-population extended haplotype homozygosity (XP-EHH) value by XP-EHH [36]. XP-EHH values were averaged over 50 kb sliding windows. These scores approximately followed a normal distribution; the threshold to locate putatively selected regions was two times the XP-EHH distribution standard deviation (|XP-EHH| > 2). Manhattan plots for F_ST_ and XP-EHH were generated with the R package gap [37].

A phylogenetic tree was generated using all variants located in these regions. Candidate genes targeted by positive selection were defined as genes overlapping with sweep regions (ZF_ST_ > 5 and |XP-EHH| > 2). GO (gene ontology) and KEGG (kyoto encyclopedia of genes and genomes) enrichment analyses for candidate genes were performed by DAVID 6.8 [38], and *p* values were corrected using the Benjamini–Hochberg method. Protein-altering mutations in these genes were listed and ranked by their single-site F_ST_ value. The protein-altering mutations of the candidate sweep gene *KDM4B* were localized to regions that are evolutionarily conserved among mammalian species.

### 2.6. Sanger Sequencing Validation

To confirm the SNPs detected in exons of the genes selected by sweep analysis, we selected eight SNPs from six genes and designed primers for their Sanger sequencing (Supplementary Table S1). Then, we outsourced the amplification and screening of SNPs in 15 monotocous and 14 polytocous sheep of this group to GENENODE (Wuhan, China).

## 3. Results

### 3.1. Sequencing and Mapping of the Sheep

We sequenced five monotocous and five polytocous sheep using an Illumina HiSeq2500 sequencer, generating a total of 627.16 million raw read pairs, comprising 180.16 Gb of raw data. After filtering out the low-quality reads, each sheep was mapped to the sheep reference genome, with an average alignment rate of 93.76% (92.79–94.34%). The percentages of reads sequenced at least once per bp varied from 87.38% to 94.55%. The percentage of reads sequenced at least four times per bp was >61.82% (Supplementary Table S3).

### 3.2. Identification of the Variation of the Sheep

We obtained about 21.39 million SNPs and 2.04 million insertions and deletions (indels) for all sheep in the two groups. Most SNPs (~71.7%) were located within intergenic regions, while only a few (~0.6%) were located within coding regions. The exonic SNPs were identified (Supplementary Table S4). There were 45,775 vs. 48,265 SNPs involving nonsynonymous mutations, 742 vs. 785 involving stop-gain variation, and 45 vs. 52 involving stop-loss nonsense variation in the polytocous and monotocous sheep, respectively. The rates of heterozygosity of the polytocous and monotocous groups were 30.02% and 30.07%, respectively. The transition-to-transversion ratios (ts/tv) were almost identical between the two groups (2.4346 for the polytocous population versus 2.4352 for the monotocous population) (Supplementary Table S5).

### 3.3. Population Structure Analysis

The levels of genome-wide genetic diversity, Tajima’s D, and the minor allele frequency (MAF) distribution indicated that high-frequency minor alleles constitute a small proportion of the total but are slightly more abundant in polytocous sheep than in monotocous ones (Supplemental Figure S1). Additionally, haplotype analysis indicated that the polytocous sheep have slower decay of pairwise correlation coefficient (r^2^) and higher integrated haplotype homozygosity (iHH) than the monotocous sheep (Supplemental Figure S2).

**Figure 1:**
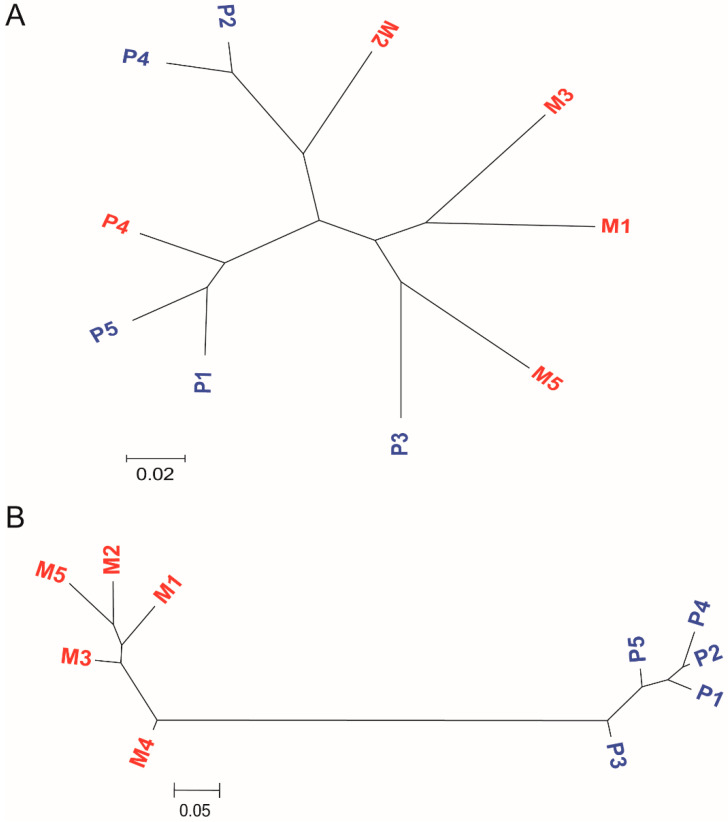
Phylogenetic and population structure of monotocous and polytocous sheep. (**A**) A neighbor-joining phylogenetic tree constructed using whole-genome SNP data. The scale bar represents the level of similarity; Monotocous (red) and polytocous (blue) samples are indicated. (**B**) A neighbor-joining phylogenetic tree constructed using SNP data selected based on F_ST_ score.

To determine the phylogenetic relationship between monotocous and polytocous sheep, a neighbor-joining tree was constructed using high-quality SNPs. When the tree was generated using the SNPs for the whole genome, monotocous and polytocous sheep formed a mixed clade (Figure 1A). This indicated that the pairwise distances within each group were larger than those between the groups and there was no significant distinction between the two groups. However, in the phylogenetic tree constructed using SNP data selected based on F_ST_ score, the monotocous and polytocous sheep were classified into two genetically different groups (Figure 1B). The results of the two trees show that the population genetic structure was not associated with the litter size and that the breeding time of high-fecundity Bamei mutton sheep was relatively short.

### 3.4. Analysis of the Selected Loci and Candidate Genes

**Figure 2:**
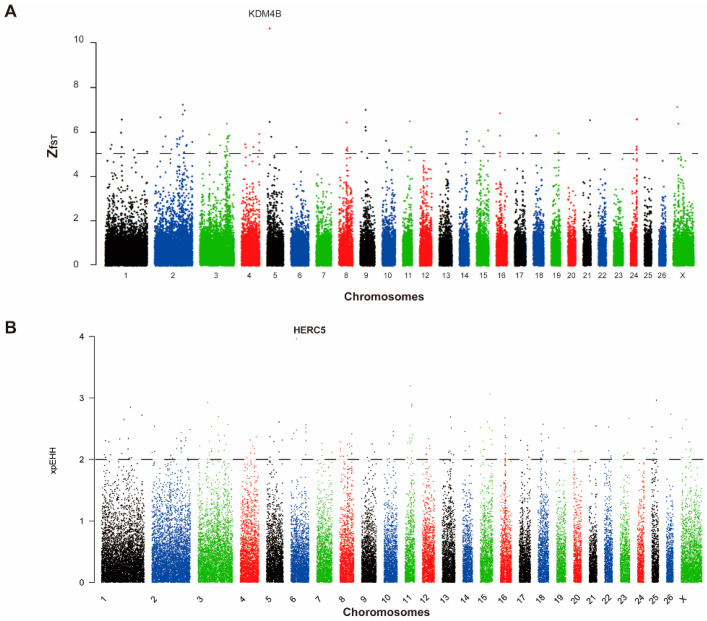
Selective sweep analysis of the monotocous and polytocous sheep. (**A**) Manhattan plot of FST among the monotocous and polytocous sheep. The FST was calculated for each 50 kb autosomal and X-chromosome window. The dashed line denotes a threshold of Z(FST) = 5; (**B**) Manhattan plot of XP-EHH among the monotocous and polytocous sheep. The XP-EHH value was calculated for each 50 kb autosomal and X-chromosome window. The dashed line denotes a threshold of |XP-EHH| > 2.

To find selection signals associated with high prolificacy, the average F_ST_ values were calculated for nonoverlapping 50 kb windows on the autosomes and X chromosome. After Z-transforming the values, we selected the windows with Z(F_ST_) > 5 across the genome, as in previous studies [34]. In total, we identified 85 unique autosomal regions and two X-chromosome regions containing 81 candidate genes. The region with the strongest selective signal (F_ST_ = 0.6639187, ZF_ST_ = 10.65708746) between the monotocous and polytocous sheep was located on chromosome 5 (16750000–16800000 bp), which contains *KDM4B* (lysine demethylase 4B) (Figure 2A, Supplementary Table S6).

We also estimated the XP-EHH statistic for the monotocous and polytocous groups using monotocous sheep as a control. An XP-EHH value greater than zero indicates that these sites have been selected in the monotocous population, while a value less than zero indicates that selection has occurred in the polytocous population. We scanned the regions using the threshold |XP-EHH| > 2 as candidate regions. A total of 198 regions including 162 genes were found to have undergone positive selection in the XP-EHH analysis, with 155 regions having undergone selection in the polytocous population. A region of chromosome 6 (36,200,000–36,250,000 bp) associated with strong selection was found to have the largest |XP-EHH| value (XP-EHH = −3.963) (Figure 2B, Supplementary Table S7).

We combined the genes obtained by the two methods described above. A total of 221 genes were found to have been selected in total. Overall, 14 genes were positively selected across both of the two methods (Supplementary Table S8).

Gene Ontology and KEGG pathway analyses were performed to further study the functions of the selected genes identified by the two methods. The enriched GO terms (*p*-value < 0.1) and KEGG pathways (*p*-value < 0.5) are shown in Supplementary Tables S9 and S10. By the Functional Annotation Clustering tool of DAVID 6.8, we identified two annotation clusters (classification stringency: Medium). Annotation cluster 2 contained the GnRH signaling pathway, estrogen signaling pathway, and oxytocin signaling pathway. The reproductive hormones in these pathways are involved in regulating sheep estrus, follicle development, and ovulation. *JUN* (*JUN* proto-oncogene, AP-1 transcription factor subunit), *ITPR3* (inositol 1,4,5-trisphosphate receptor type 3), and *PLCB2* (phospholipase C beta 2) were enriched in all three pathways (Table 1).

### 3.5. Mutations in the KDM4B Gene

**Figure 3:**
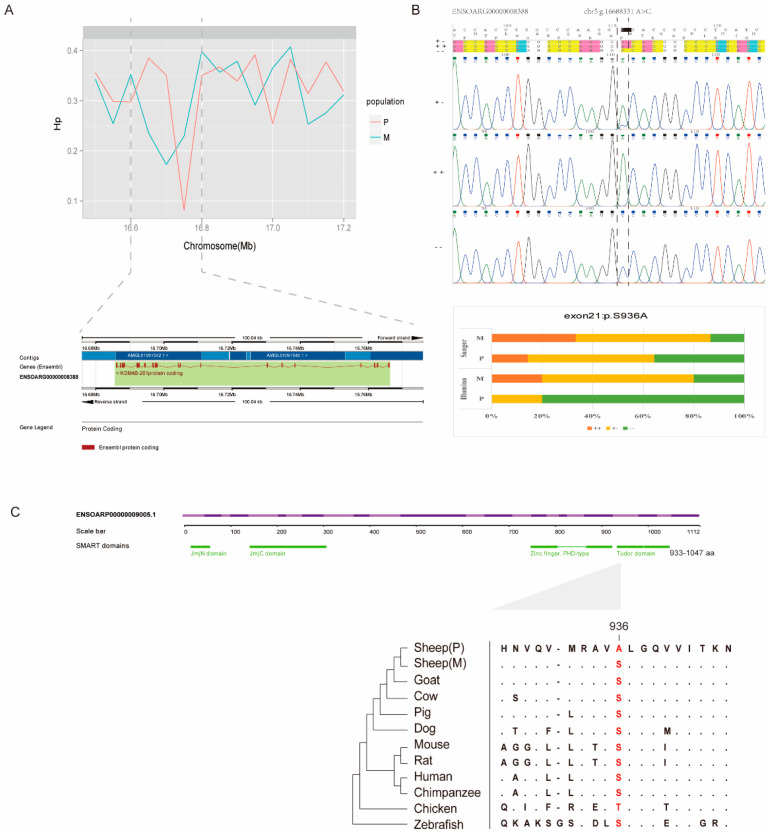
*KDM4B* mutation in the coding region. (**A**) Hp value around the *KDM4B* loci. Hp was calculated for each 10 kb window of the monotocous and polytocous sheep. The gene coordinates are based on Ensembl ID ENSOARG00000008388; (**B**) Percentages of homozygotes and heterozygotes in the monotocous and polytocous sheep. The reference and mutant alleles are represented by “+” and “−,” respectively. Besides the 10 sheep sequenced with Illumina technology, additional samples were genotyped by Sanger sequencing; (**C**) Evolutionary analysis of the p.S936A amino acid variant. The protein coordinates are based on Ensembl ID ENSOARP00000009005.1. The upper panel shows the domains of the protein. The orthologous protein sequences from 11 vertebrates are aligned with the mutant residues shown in red. The NJ tree derived from the multiple alignment is shown in the lower panel.

*KDM4B* is located in the highly differentiated region with the highest F_ST_ value between the monotocous and polytocous groups. A selected mutation that goes to fixation tends to reduce variation in linked sites in the process of a selective sweep [39]. Therefore, we determined the Hp value of the window (chr 5: 16,750,000–16,800,000 bp) around *KDM4B*. The Hp value of the polytocous group (Hp = 0.0818) decreased in the *KDM4B* region and was the lower than in the monotocous group (Hp = 0.22923) (Figure 3A).

To identify SNVs subjected to selection, we screened the exonic mutations of the *KDM4B* gene in both monotocous and polytocous groups. SNVs that can alter protein translation, structure, and even function may contribute to rapid evolution in domestic animals [40]. Here, 13 synonymous SNVs, 3 nonsynonymous SNVs, and 1 frameshift deletion were identified. All of the three nonsynonymous SNVs cause amino acid sequence changes *p*.S570G (F_ST_ = 0.11), *p*.S924L (F_ST_ = 0), and p.S936A (F_ST_ = 0.45) in the translated protein (in accordance with Ensembl gene annotation) (Supplementary Table S11).

We compared the F_ST_ values of the three nonsynonymous SNVs; only mutation p.S936A had high divergence of allele frequency within our population: monotocous sheep 50% (*n* = 5) and polytocous sheep 90% (*n* = 5). To confirm these frequencies based on Illumina sequencing, alleles of additional samples were genotyped by Sanger sequencing. The frequency of mutant allele of *KDM4B* gene genotyped by Sanger sequencing showed a slight decrease in polytocous group 60% (*n* = 14), compared with the result in whole genome sequencing. The mean of individual litters variants in polytocous sheep may affect the distribution of the genotype when increasing number of samples. The frequency of the mutant homozygote in polytocous sheep was higher than that in monotocous sheep. Moreover, the distribution of KDM4B p.S936A genotype contained significant differeces between polytocous and monotocous sheep population genotyped by Sanger sequencing (*p*-value = 0.041). (Figure 3B). This indicates that the nonsynonymous mutations may be associated with the selective sweep at *KDM4B*.

p.S936A affected the TUDOR domain (Figure 3C). This domain can bind to specific lysine methylation marks on histone proteins (H3-K4me3, H3-K23me3, and H4-K20me3). It plays a vital role in chromatin localization and the regulation of enzymatic function [41]. Thus, we aligned the *KDM4B* protein mutant with its ortholog in diverse vertebrates to evaluate the functional effects of the variants. The results reveal that p.S936A is quite well conserved, being invariant among all of the other mammals that we used (Figure 3C). All of the results indicate that p.S936A is an important mutation for the reproduction-related *KDM4B* sweep.

## 4. Discussion

Continuous artificial selection in production-oriented breeding has left selective signatures and genomic variability in domesticated sheep. In this study, we performed whole-genome sequencing of 10 Bamei mutton sheep with different litter sizes from the same population. In this population, reproductive traits have undergone intensive selection via the breeding strategy. In this research, numerous mutations were selected in the population after screening, but the rates of heterozygosity did not differ between the two populations. When a neighbor-joining tree was constructed using SNPs selected based on F_ST_ score, these two groups were differentiated into two genetic clusters. Most quantitative traits are known to respond quickly to artificial selection, and this population subjected to selection of litter size might have evolved in opposite directions with soft sweeps [42,43].

Selective sweeps can identify important genomic regions that have been swept in the recent past. Some genes associated with complex, economically important traits have been identified with the help of selected sweep analysis by whole-genome sequencing. We used the XP-EHH test to detect alleles near fixation within a Bamei mutton sheep population. Upon undergoing recent selection, the selected allele generally reaches a high frequency or fixation in one group, but remains polymorphic in the whole population [44]. In this research, we discovered 155 regions selected for in the polytocous population, which was greater than the 43 regions screened in the monotocous population. Using fixed window selection of F_ST_ and XP-EHH, 221 candidate genes were found to be associated with the prolificacy trait. In a previous study using a segregated flock based on QTL and GWAS mapping, some mutations (FecB, FecX, and FecG) were identified to affect ovulation in sheep [11]. However, here we did not identify any mutations previously reported to increase the number of ovulations in sheep. The litter size per breeding ewe is not only influenced by ovulation, but also affected by a number of factors including fertilization rate and pregnancy loss (in the embryonic and fetal development period). Recently, the *LEPR* gene, estrogen receptor 1 (*ESR1*) gene, and prolactin (*PRL*) gene were found to be associated with fecundity, as revealed by a selective sweep analysis in European commercial and semi-feral breeds and a Chinese indigenous breed distributed in different ecoregions [23,45,46].

*JUN*, *ITPR3*, and *PLCB2* in the selected region were enriched in gonadotrophin releasing hormone (GnRH), oxytocin, and estrogen signaling pathway associated with the complex process from estrus to lambing. This process is organized by complex communication among the hypothalamus, pituitary, ovary, and uterus. GnRH, secreted by the hypothalamus, regulates the synthesis and release of gonadotrophins, follicle-stimulating hormone (FSH), and luteinizing hormone (LH) from the pituitary [47]. FSH and LH support the growth and maturation of follicles. Estradiol and progesterone secreted by the corpus luteum inhibit GnRH release by feedback modulation. During the follicular phase, following luteolysis, estradiol reaches a critical threshold and stimulates the preovulatory gonadotrophin surge, appearing to help the ovulation of mature follicles [11].

PLCB is involved in a wide range of signals of reproductive processes, being regulated by many hormones (FSH, LH, GnRH, oxytocin). *PLCB2* as a member of the PLCB subfamily is activated by G-protein-linked receptors and can hydrolyze phosphatidylinositol 4,5-bisphosphate to form inositol-1,3,4-trisphosphate (IP3) and diacylglycerol (DAG), which stimulate Ca^2+^ release and protein kinase C activity, respectively [48]. *ITPR3* is a member of the IP3 receptor family. IP3, as an intracellular secondary messenger, mobilizes Ca^2+^ from endoplasmic reticulum stores, which transduces several calcium-dependent cascades. IP3 receptor downregulation induced by GnRH can suppress the secretion of LH/FSH [49,50].

*JUN* is one of three *JUN* members [*JUN* (c-*JUN*), *JUNB*, and *JUND*] constituting the AP-1 family of heterodimeric transcription factors by combining with four FOS members [FOS (c-Fos), FOSB, FRA1, and FRA2]. GnRH can stimulate the expression of *JUN* by rapidly and transiently binding to the AP1 site in the *FSHB* promoter and then stimulating *FSHB* transcription [50]. Conditional knockout of *JUN* in mice resulted in subfertility in both sexes, such as impaired spermatogenesis in males, diminished corpora lutea in females, and lower gonadal steroid (GnRH and LH) levels [51].

The nonsynonymous mutation p.S936A at *KDM4B* was discovered and confirmed in monotocous and polytocous sheep; it may be the reason for the signal of a selective sweep at the *KDM4B* locus. *KDM4B* belonging to the KDM4/JMJD2 family of histone demethylases contains a JmjN domain, JmjC domain, tandem plant homeodomains (PHD), and tandem Tudor domains. *KDM4B* catalyzes the demethylation of H3K9me3 and H3K9me2 at or near regulated promoters to promote expression of the downstream pathway induced by multiple different extracellular stimuli [52]. *KDM4B* plays a central role in regulating the Estrogen Receptor (ER) signaling cascade by controlling expression of the ER and FOXA1 genes. These two important genes can maintain the estrogen-dependent phenotype [53]. In the developing ovarian follicle, granulosa cells are the main producers of estrogen. *KDM4B* is expressed in granulosa cells at early stages of folliculogenesis and its level is correlated with pregnancy failure in IVF patients [54]. *KDM4B* expression in the uterus is also associated with recurrent pregnancy loss in women [55]. The identified intersection between steroid hormones and *KDM4B* in the ovary and uterus sheds new light on the regulation of reproduction.

## 5. Conclusions

Following high-throughput sequencing, SNPs and indels were identified from two sheep populations. The sequencing data revealed the genetic diversity and population differentiation of the Bamei mutton sheep population experiencing selection for litter size. The genomic selection scan detected some interesting candidate genes and pathways under artificial selection, which might have increased litter size. This genome-wide research provides valuable information for future whole-genome selection for fecundity in this sheep breed.

## Figures and Tables

**Table 1:** The functional annotation clusters of similar biological meanings sharing common gene members enriched by DAVID.

**Annotation Cluster 1**	**Enrichment Score: 1.95**			
**Category**	**Term**	**Count**	**Genes**	***p*-Value**
KEGG_PATHWAY	oas04660:T cell receptor signaling pathway	8	*CD3D CD3E CD3G JUN MALT1 CARD11 MAP3K14 PAK6*	1.35 × 10^−5^
KEGG_PATHWAY	oas05142:Chagas disease (American trypanosomiasis)	5	*CD3D CD3E CD3G JUN PLCB2*	0.013
KEGG_PATHWAY	oas05166:HTLV-I infection	6	*ATM CD3D CD3E CD3G JUN MAP3K14*	0.072
KEGG_PATHWAY	oas05162:Measles	4	*CD3D CD3E CD3G ADAR*	0.094
KEGG_PATHWAY	oas04640:Hematopoietic cell lineage	3	*CD3D CD3E CD3G*	0.157
**Annotation Cluster 2**	**Enrichment Score: 0.67**			
**Category**	**Term**	**Count**	**Genes**	***p*-Value**
KEGG_PATHWAY	oas04912:GnRH signaling pathway	3	*JUN ITPR3 PLCB2*	0.151
KEGG_PATHWAY	oas04915:Estrogen signaling pathway	3	*JUN ITPR3 PLCB2*	0.188
KEGG_PATHWAY	oas04921:Oxytocin signaling pathway	3	*JUN ITPR3 PLCB2*	0.340

## Data Availability

The raw genomic sequencing data from this work have been uploaded to the NCBI SRA database with accession number PRJNA560632 (https://www.ncbi.nlm.nih.gov/sra/PRJNA560632). The variation data reported in this paper has been deposited in the Genome Variation Map [56] in National Genomics Data Center [57], China National Center for Bioinformation/Beijing Institute of Genomics, Chinese Academy of Sciences, under accession number GVM000114 at http://bigd.big.ac.cn/gvm/getProjectDetail?project=GVM000114.

## Supplementary Materials

The following are available online at https://www.mdpi.com/2076-2615/11/1/157/s1, Figure S1: Distributions of (A) minor allele frequency and (B) Tajima’s D across the genome of Monotocous (Blue) and polytocous (Red), Figure S2: Levels of linkage disequilibrium across the genome of Monotocous (Blue) and polytocous (Red), Table S1: Primer information of Sanger sequencing, Table S2: Sample information and sequencing quality, Table S3: Reads mapping statistics and coverage of depth for Monotocous and Polytocous sheep, Table S4: Number of SNPs and Indels in different chromosomes, Table S5: Number of annotated SNPs in different gene regions, Table S6: Genomic regions and genes highly differentiated (Z(FST) > 5) between Monotocous and Polytocous sheep, Table S7: Candidate regions and genes of 50-kb windows with value of |XPEHH| > 2 in Monotocous and Polytocous sheep, Table S8: Overlapping selected windows selected by FST and XP-EHH, Table S9: Gene Ontology enrichment analysis of the 221 candidate genes with GO terms (*p* < 0.1), Table S10: Ranking of KEGG pathway with Pvalue (*p* < 0.5) enriched from 221 genes, Table S11: Mutations in exons of KDM4B gene.
