# Supplementary material for: Whole Genome Sequencing Reveals the Effects of Recent Artificial Selection on Litter Size of Bamei Mutton Sheep
Source: Animals (Basel). 2021 Jan 12;11(1):157. doi: 10.3390/ani11010157 (PMC7827510; doi:10.3390/ani11010157)
Supplement: Supplementary file 1 [file animals-11-00157-s001.zip › Supplemental tables and figures/Supplimental Figures.pdf]

Whole Genome Sequencing Reveals the Effects of Recent Artificial Selection on Reproduction Trait of Bamei Mutton Sheep

Yaxin Yao[1], Zhangyuan Pan[1], Ran Di[1], Qiuyue Liu[1], Wenping Hu[1], Xiaofei Guo[1,2], Xiaoyun He[1], Shangquan Gan[3], Xiangyu Wang[1,*], Mingxing Chu[1,*]

Supplementary Figures

Figure S1……………………………………………………………. 2

Figure S2……………………………………………………………. 3

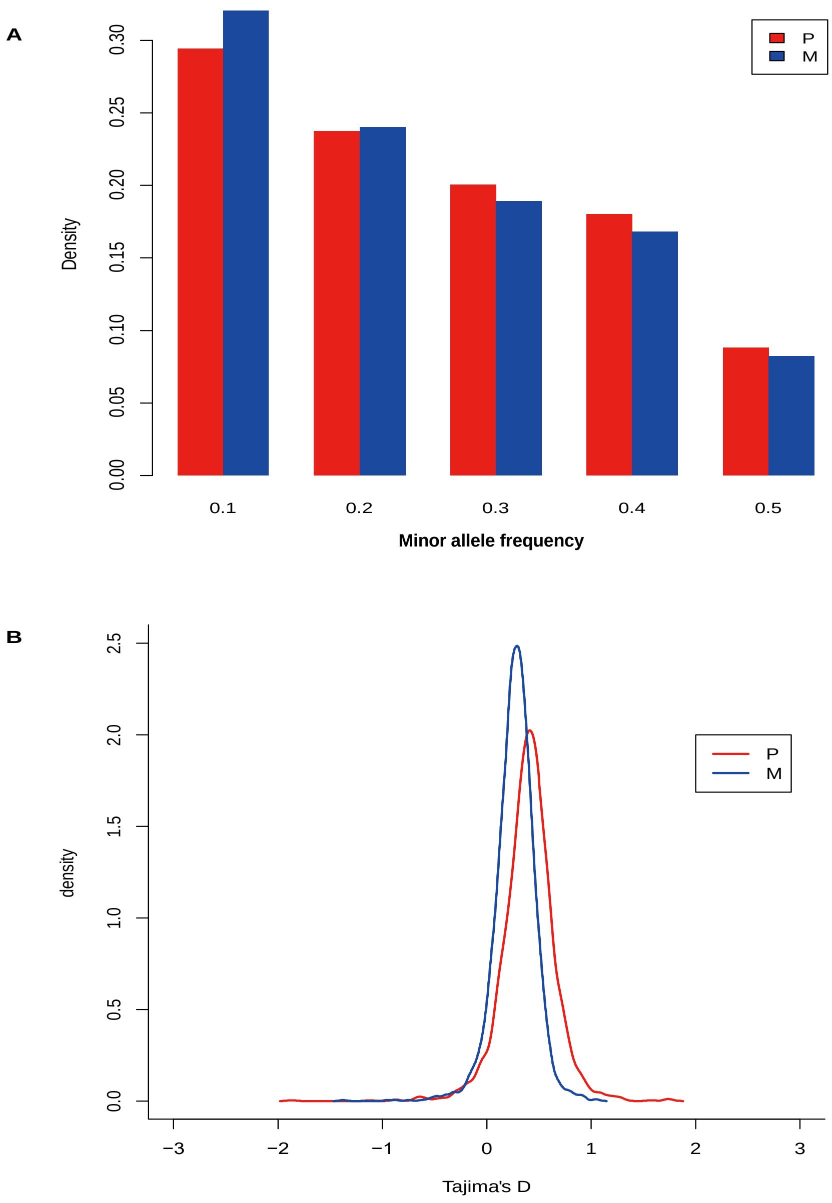

Figure. S1. **Distributions of (A) minor allele frequency and (B) Tajima's *D* across the genome of Monotocous (Blue) and polytocous (Red).**

Alleles with frequency lower than 0.10 were not considered because of the insufficient sample size. The Tajima’s *D* was calculated for a 50 kb sliding window along the genome.

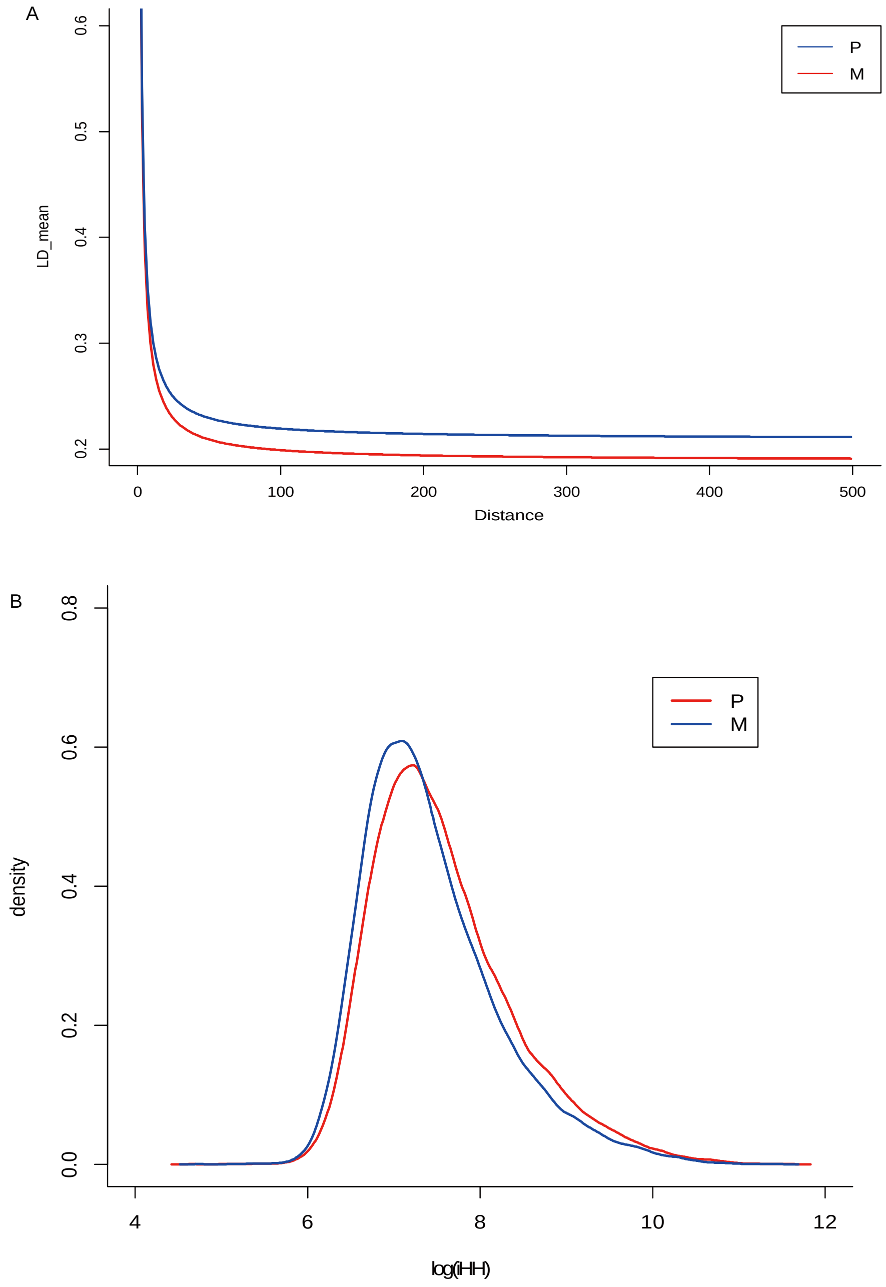

Figure. S2. Levels of linkage disequilibrium across the genome of Monotocous (Blue) and polytocous (Red).

(A) The correlation coefficient (r^2) between two markers decays with the increase of their pairwise distance. The average r^2 against distances was plotted using lowess regression in R. (B) Distributions of integrated haplotype homozygosity (iHH). The iHH was calculated for a 50 kb sliding window along the genome.
